# Implementation of UN Convention on the Rights of Persons with Disabilities in public and private schools in three districts of Uganda

**DOI:** 10.4102/ajod.v11i0.908

**Published:** 2022-10-27

**Authors:** Elijah Musenyente, Marie L. Han, Michel Knigge

**Affiliations:** 1 Department of Community and Disability Studies, Faculty of Special Needs and Rehabilitation, Kyambogo University, Kampala, Uganda; 2 Department of Pedagogy, Faculty of Human Sciences, University of Potsdam, Potsdam, Germany; 3 Department of Rehabilitation Sciences, Humboldt-Universitaet zu Berlin, Berlin, Germany

**Keywords:** people with disabilities, UN Convention, inclusive education, disability rights, Uganda

## Abstract

**Background:**

The study was grounded in the recent developments of implementing the United Nations Convention on the Right of Persons with Disabilities (UNCRPD) in schools in Uganda, leading to a renewed interest in the questionings about inclusive education.

**Objectives:**

The inclusive approach was evaluated in terms of: (1) how public or private schools in Uganda understand inclusive education; (2) how schools implement inclusive education under the influence of the UN Convention; and (3) what determines the course of action and school routine of private and state schools.

**Methods:**

This exploratory qualitative research included interviews with six schools purposively selected in Mukono, Wakiso and Kampala districts of Central Uganda. The selected schools comprised three private and three state schools (i.e. representing primary and secondary schools and a vocational training institute).

**Results:**

The research demonstrated that ‘inclusive education practice’ that was upheld by all the schools, was ironically stained with exclusion, for example, by non-admission of students with visual and hearing impairment, inaccessible physical environment, inadequate funding and separation of students according to abilities. However, whilst all schools followed the regular curriculum, some schools developed their own ways of teaching learners with diverse learning needs.

**Conclusion:**

Some schools in Uganda have developed meaningful approaches of including students with disabilities but there are still many challenges for many. Enforcing Uganda’s disability policies adapted from the CRPDs could enable learners with disabilities to enjoy their legal rights.

**Contribution:**

Since the intention of inclusion of students with disabilities stands in contrast to the reality of practice found in many schools, there seems to be a need to carry out regular assessments and measures of support for a sustainable inclusive school development.

## Introduction

Uganda’s ratification of the United Nations Convention on the Rights of Persons with Disabilities (UNCRPD) on 25 September 2008 (UNCRPD 2010; United Nations 2016) ‘Concluding observations on the initial report of Uganda’ provided an approach and direction of inclusive education within the education system. The system considers the general education of students with disabilities, which enable or aggravate good-quality inclusive education. Article 24 (2)-Education, of the UNCRPD (2006) provides that:

Persons with disabilities are not excluded from the general education system on the basis of disability, … free and compulsory primary education, or secondary education, on the basis of disability; … can access … on an equal basis with others in the communities in which they live; … receive the support required, within the general education system, to facilitate their effective education. (p. 17)

Many African countries and other states around the world appended their signatures on the UNCRPD and have been debating on the notion of ‘inclusive education’ in an African context and different ideas have come up (Mahlo 2017). This new concept originated from failure by community to recognise individual difference as an important factor in the education and schooling of learners with severe learning disabilities or special education needs who sometimes require individualised assistance.

By signing the UNCRPD, Uganda consented to fully support those members of society, who have any kind of disability and agreed to stand up for their rights. The convention is geared towards all areas of life and encourages the relevant group of persons to be able to fully take part in it, whereby this study specifically concentrated on the right to education for children and young adults with special needs.

Primary education in all public schools in Uganda is free of any sort of tuition fees since 1997, which expanded towards free secondary education 10 years later (Mwesigwa 2015). Every child in the state of Uganda therefore has the right to education and in conjunction with the UNCRPD, regardless of different health-related backgrounds, should be able to get support in attending school at any time. However, a clear distinction between private and public schools in Uganda has to be made. About one-third of all primary schools and two thirds of all secondary schools belong to the private sector, which means that they require some sort of financial contribution from the students’ families (Initiative for Social and Economic Rights [ISER] 2015). As private institutions cover such a large portion of the educational system in Uganda, they play a main role in practicing inclusive education, operating on different levels when compared with the financial foundations of state schools.

The Ministry of Education and Sports (MOES) within the Ugandan government developed both a curriculum, which was released in 2007 and is still relevant, as well as the most recent Education and Sports Sector Strategic Plan, which covers the time period 2017–2020 (MOES 2017). Both the curriculum and the Sector Strategic Plan determine ways of operations and standards for schools in Uganda to follow and accomplish. These documents are profoundly following the models of the UN Convention, setting the bars high for high-quality and inclusive education.

### Education system

The Ugandan education system follows the British academic model. A school year is divided into three terms of three months with a holiday break of a couple of weeks in between (MOES 2021). Schooling starts on the pre-school level, in nursery. Attending nursery from age three to six is much more popular in bigger cities than in villages. Here, the educational path mostly starts with primary school (Hands 4 Uganda 2018).

Primary school begins about the age of six and continues for seven years, until pupils reach the age of around 13. The first major national exam – the Primary Leaving Examination (PLE) – is taken at the end of Primary 7. The subjects English, mathematics, social studies and science need to be passed in order to attend secondary education (World Education Services [WES] 2020). After passing the PLE, the decision is made of either attending secondary school or some kind of vocational education usually offered in the form of craft courses in technical schools (Kim 2021).

Secondary schools, on the other hand, continue for six years and are attended by students from the age of about 13 to 19 years. The first stage is called the O-level period, which lasts for four years, from Senior 1 to Senior 4 and is completed by the second major national exam, the Uganda Certificate of Education (UCE) or simply the O-level examinations. By passing the UCE, the students obtain the chance to attend the second stage of secondary school, called A-level period, for two years from Senior 5 to Senior 6. Passing the final exam at the end of Senior 6, the so-called A-levels or Uganda Advanced Certificate of Education (UACE), qualifies the students to attend university (WES 2020).

The African Union (2014) advised that high tuition fees prevented some children from being sent to school by their parents. Accordingly, in 1993, the Ugandan government decided to liberalise the education sector, and although primary education in public schools is free of tuition many Ugandan families, mostly in the rural areas, still cannot afford sending their child to school. They do not have enough money to pay for the school materials, such as pens and books, the uniform or even school lunches, which they generally have to cater for themselves (Mwesigwa 2015). Whilst inclusive education is allocated a small budget in many African countries, Zubairi and Rose (2019) argued that sub-Saharan Africa government’s expenditure on secondary and primary education is too little compared with anywhere else on the globe. For example, public primary and secondary schools in Uganda claim not being able to provide an adequate school environment to cater for all children because of lack of adequate funding, the private schools keep increasing the tuition fees in order to improve the quality of education (Mutebe 2017). The for-profit private schools, with medium to high tuition claims, are fully financed from private sources most of the time, whereas the non-profit private schools often depend on the tuition fees to offer an appropriate quality of education (ISER 2015).

### Ugandan Special Needs and Inclusive Education Department

The Ugandan Special Needs and Inclusive Education Department forms part of the MOES. It is responsible for giving out technical and practical guidelines to schools and other educational establishments to follow the inclusive education standards. By signing the Salamanca Statement and Framework for Action on Special Needs Education in 1994, Uganda agreed to focus on the inclusion guidelines. Under those guidelines, the department developed operating principles and objectives in order to meet the set standards thus: ‘It is all about changing attitudes, behavior, teaching methods, curriculum, environment and allocation of human, material and financial resources to meet the educational needs of all learners’ (MOES 2018).

### Statement of the problem

Although there has been some progress made across African countries (including Uganda), inclusive education has not yet been achieved because of several challenges that include the allocation of inadequate funds for education (Chitiyo & Dzenga 2021:61). When Uganda signed the UNCRPD in 2008, it consented to fully support those members of society, who have any kind of disability and agreed to stand up for their rights. Unfortunately, the concrete strategies that were intended to include a wide variety of students by focusing on their specific individual needs under different school environments actually stand in high contrast to the reality. On average only one out of four students is able to complete all levels of primary school, which results into a very high primary school dropout rate (United Nations Development Programme 2016). Besides the fact that the curriculum and the UN Convention both became effective over a decade ago, the present state of Ugandan schools is fragile when looking at the number of students who are not able to attend or complete primary education. That leads to the conclusion that there is some sort of barrier between the actual school practice and the governmental standards and directions. Despite the matter being of such high importance, actuality and affecting a huge part of the overall population, there is a significant lack of research on the topic of the establishment of inclusive education in Uganda. Thus, the article seeks to address those issues.

### Aim

The study explored the implementation of inclusive education by private and state schools in Uganda under the influence of the UNCRPD and provided recommendations based on informed research.

### Research questions

1. How do public and private schools in the districts of Mukono, Wakiso and Kampala understand inclusive schools and classrooms?
2. How do Ugandan private and state schools in the districts of Mukono, Wakiso and Kampala implement high-quality inclusive education amongst a diversity of learners as per the curriculum and strategic plan under the influence of the UN Convention?
3. What determines the course of action and school-routines of private and state schools in the districts of Mukono, Wakiso and Kampala?

## Materials and methods

### Area description

Uganda is a presidential republic located in East Africa, on the edge of the Equator. Bordered by the Democratic Republic of Congo, Kenya, Rwanda, Sudan and Tanzania, Uganda accommodates 39 million people on 241 038 km^2^ (World Atlas 2019). Kampala lies in central Uganda and is the largest and capital city of Uganda with an estimated population of 1 680 800 people as of 31 July 2019; its metropolitan area include Wakiso District, Mukono District, Mpigi District, Buikwe District and Luweero District has an estimated population of 6 709 900 people in 2019 in an area of 8451.9 km^2^ (3263.3 square miles [sq mL]) (Uganda Bureau of Statistics 2019).

### Participants

All the six schools were located around the districts of Kampala, Mukono and Wakiso districts in central Uganda, thus making them comparable. However, the limited sample of six interviews with principals and teachers was not considered to be representative of the whole country. The duration of the interviews with principals and teachers varied from 45 min to just over an hour. In order to put the conversations onto paper afterwards, all of the principals and teachers interviewed agreed on being voice-recorded. Often, more than one person from each of the school was part of the discussion, giving rise to the possibility to capture multiple points of view.

Part of visiting the six schools was also getting a closer look at the circumstances and viewing the facility, to put things into perspective and understanding the circumstances students and teachers were faced with in their day-to-day school life. Experiencing those vocational contexts made it possible to contrast the different school standards and provided space to speculate about the management strategies and choices. Every school was assigned an individual code for retaining anonymity whereby the first letter within the code referred to being either a private (*P*) or a state institution (*S*), whereas the second two letter correlated with the school type (*PS* for primary school, *HS* for high school and *Voc* for vocational training) as shown in Table 1.

**TABLE 1:** A description of schools that participated in the study.

No. of schools	Category of learners	School category	Participant position	Gender	Identifier code
1	All learners	Private primary school (PPS)	Director (D)	Male (M)	PPS, D, M
1	All learners	State primary school (SPS)	Head teacher (H)	Female (F)	SPS, H, F
1	All learners	State high school (SHS)	Head teacher (H)	Male (M)	SHS, H, M
1	All forms of disabilities e.g., physical, hearing, visual, intellectual, multiple disabilities etc.	State vocational training school (S-Voc)	Manager (M)	Female (F)	S-Voc, M, F
1	Learners with physical disabilities	State-aided special primary school for children with physical disabilities (SPS-SEN)	Head teacher (H)	Female (F)	SPS-SEN, H, F
1	All forms of disabilities e.g., physical, hearing, visual, intellectual, multiple disabilities etc.	State-aided special primary school church founded (SPS-SEN-CF)	Head teacher (H)	Female (F)	SPS-SEN-CF, H, F

### Methods

This was an exploratory qualitative study, which was grounded in the views and experiences of principals and teachers of both private and public schools in the area of Kampala, Mukono and Wakiso districts in central Uganda from March 2017 to February 2018. A purposive sampling technique was used for selecting the sample of principals and teachers of six schools, which claimed to practice inclusiveness, that is, three private schools and three public schools. The purposeful sampling technique was both cost-effective and time saving and was basically chosen by the investigators basing on prior knowledge and mindful of the purpose of the study of inclusive education by key players (i.e. principals and teachers).

### Data collection

Data were collected using semi-structured interviews. The use of open-ended questions was of particular importance because the participants got the feeling of being expected to answer in a certain way because of one’s own values. This method enabled an honest and open exchange, making it possible to work out important perspectives for the research (Busetto, Wick & Gumbinger 2020).

For the textualisation of the spoken information and communication later on, the participants were informed in advance about recording the process.

Content analysis was used to analyse data. Data were subdivided into five steps starting with the development of analysis categories based on the collected material. The central topics within the interviews were taken into account whilst focusing on the concrete statements of the participants. As a next step, all significant categories of the various interviews were compiled and where needed, augmented. This step was followed by the coding of the material. Each valid statement was assigned to a specific analysis category, which reduced the amount of information from all the interviews and emphasised the essential parts (Tenny et al. 2021).

### Ethical considerations

The study was reviewed for ethics and scientific merit and received approval by an accredited ethics committee in Uganda, The Acquired Immunodeficiency Syndrome (AIDS) Support Organisation Research and Ethics Committee (Ref: Protocol: # TASOREC/109/2020-UG-REC-009). Informed consent was obtained from the participants before conducting the interviews.

## Results

### How public and private schools understood inclusive schools and classrooms

This study explored the teachers’ understanding and knowledge concerning what is involved in inclusive education, for example, their attitudes regarding inclusive education; skills and capacity in integrating inclusive practices; the collaboration with parents who are better well versed with the needs of their children, provision of accessible physical environment and the funding. The results were as follows:

#### Admission and accommodation of diversity (every child)

The way different schools in the districts of Mukono, Wakiso and Kampala interpreted and practiced the notion of inclusion and inclusive education strongly varied according to their school concept and the students attending the school. Some of the institutions specialised according to their individual school description in admitting and educating students with special needs. Other schools were more challenged and struggled with accommodating every child, as they did not have the relevant knowledge and resources. The admission process was one of the fundamental parts of inclusiveness and usually started with the assessment of the students in state schools as follows:

‘Within the school level here … have a board which does the assessment. We assess and determine the problem of the learner. So when he is in primary seven that’s when we inform the National Examinations Board in writing and they send someone who can assess.’ (SPS-SEN-CF, H, F)

However, the assessment of students in vocational training institutes was slightly different as it involved a medical doctor from a public hospital as indicated:

‘Because with certain disabilities, you could not be allowed to do every course you want. So require them assessed in a government hospital, also to let us know any other kind of illnesses that may need special attention, when here.’ (S-Voc, M, F)

Whilst three of the six schools in the study embraced inclusiveness, they still institutionalised students with disabilities although they did not support the idea thus:

‘If we are institutionalising them, then we are openly coming out … I don’t think, it is a good thing.’ (S-Voc, M, F)

Not accommodating students with severe visual impairments is what all of the schools had in common because some students with disabilities were sent to attend special schools where they received the attention and support needed to participate in education. All the schools in the study did not have the resources to reformat the school materials or to hire special teachers to provide for blind students and therefore could not admit students with visual impairment thus:

‘They are those who cannot see, the blind ones, right now we don’t admit them. There is a school somewhere [*at Salama School for the Blind*] which accommodates all the blind. So the rest we can accommodate.’ (SPS-SEN-CF, H, F)

‘If someone wants to take a course and he’s blind, we have to turn him down.’ (S-Voc, M, F)

When it came to students with hearing impairments, it often depended on whether they were able to use the appliances, which allowed them to hear. Some of the interviewed schools admitted students with hearing impairments who were hard of hearing and made the effort to cater for them especially by allowing them to sit near the teacher and using assistive hearing devices. Beyond that, students with hearing impairments were referred to the school for the deaf where they used sign language thus:

‘We admit the children … Physical and learning difficulties. When a deaf child comes, we do not admit him or her. Instead, we refer him or her to other schools.’ (SPS-SEN, H, F)

Students with hearing and visual impairment were excluded and treated in almost similar manner because they attended special schools where separate educating methods and tools were used and most of the schools lacked them:

‘No, we don’t have the deaf and we don’t have the blind.’ (SPS-SEN, H, F)

All of the schools, which claimed to be inclusive including the Vocational Rehabilitation Center, admitted students with intellectual impairments and learning difficulties, including dyslexia, down syndrome, autism, etc. Some students had a lot of learning problems but in different levels. Some could learn but at a slow pace thus:

‘Like we have mild, severe and profound.’ (SPS-SEN, H, F)

Students with learning difficulties got admitted as they were able to learn with other students although they needed more assistance. However, other state schools were very strict during the assessment and admitting of students as follows:

‘They rank us highly, we have to perform better than the others in the area. So you have to sit for an interview and if you perform better, you can go to P3, for example. But if you fail, we place you where you are fitting.’ (SPS, H, F)

Where the environment was well adjusted, all the schools accommodated students with any kind of physical disabilities. They were able to fully partake in everyday school life on an intellectual level although some of them lacked assistive devices and experienced inaccessible physical environment such as stepped entrances and inaccessible toilets. However, one special school for students with physical disabilities had an accessible physical environment as reported:

‘For example those ones in wheelchairs, us, we do accommodate them.’ (SPS-SEN-CF, H, F)

Schools were faced with a rethink when transforming into an inclusive and open-minded institution, as they needed to get more considerate and took several more aspects into account in order to provide for the wide variety of students as follows:

‘Inclusion, so we tried, but I don’t know if I’d call it luck, but we don’t have many children with special needs.’ (PPS, D, M)

#### Barrier-free physical accessible school environment

Creating an inclusive and safe accessible school environment involves a barrier-free physical infrastructure. Students with physical impairments, who use wheelchair or crutches for mobility were taken into consideration to show some inclusion, for example, when constructing the buildings as they needed to be able to move freely and independently. Access ramps were constructed in the two schools, which were founded on the grounds of disability:

‘We even have toilet facilities for people, who are disabled.’ (SHS, H, M)

However, most state schools had a few ramp access on recently constructed buildings whilst toilets had stepped access and narrow entrances. Although the highest number of students at the vocational training school had physical disabilities, the school’s roads and paths were inaccessible as per the following exclamation:

‘When you talk about accessibility I want to get off my chair and hide under the table. Seriously, because we preach about it, we talk about it everywhere, and we’re the ones, who are supposed to have out places accessible for people with disabilities. But as you were driving through our road, you saw it!’ (S-Voc: 158–163)

#### Attitude

In addition to struggling with prejudices and other difficulties in their everyday school life students with disabilities experienced pressure to achieve the set goals. This was even more conflicting when repeating a class was weighed down with negative connotations instead of seeing it as a chance to improve the knowledge or skills in certain fields as expressed here:

‘When I have a child who has to repeat Primary 5 three times and cannot write his name, count up to 100, add up to 20 … This child cannot do more than Primary 5, just Primary 1 to 4.’ (SPS-SEN-CF, H, F)

#### Assistive devices

The schools that were founded on the basis of disability tried to solicit for assistive devices for their students with special needs as follows:

‘Most times we collaborate with partners … Orthopedic workshops and we get some devices … wheelchairs.’ (S-Voc, M, F)

#### Collaboration with parents

Collaboration between teachers and parents permitted the sharing of experiences for the sake of understanding the student’s needs, well-being and learning most especially, learners with special educational needs. The schools used effective and respectful communication to parents, putting in mind the language, cultural background and socioeconomic status of the parents. Collaboration was in the form of monitoring the educational progress and joint decision on the class level and vocational programme for the students. This was evident from the statement:

‘You talk to the parents … about a, b, c, d and so on and what the problem is. You try several approaches to see, what the problem is.’ (SPS-SEN-CF, H, F)

#### Funding for schools

In each school, the teaching and learning environment strongly depended on the source and the management of the given financial resources. Except for the private institutions, most of the schools in the study were given specific funding by the government. This amount was intended to pay a set and standardised number of teachers and the basic learning materials. This funding was usually based on the number of students in the school. However, government funding was inadequate to cater for the needs of state schools as indicated here:

‘This school needs a lot of people to work here. We need therapists, we need social workers and who is willing to pay them? The government is not financing these people I am talking about … we are 42 staff members, and the government is paying only 13 teachers.’ (SPS-SEN, H, F)

There were two ways of seeking funding for schools as guided by the public authority or the private sector. Primary education in all public schools in Uganda is free of any sort of tuition fees, and therefore, state schools had to rely on the approval of financial funds by the government, which was also inadequate as follows:

‘State schools have … challenges, because they are told not to charge the students any money. And yet the money, they get from the government is very little. For every student in primary school, the government pays … less than one dollar per term.’ (PPS, D, M)

However, all the state schools in the study asked for some sort of contribution from parents to pay for their children’s lunch. Private schools required parents or sponsors of the students to contribute to the financial means by paying a certain amount of tuition. These private schools were struggling to meet their operation expenses such as payment of teachers’ salaries, water and electricity bills, teaching and learning materials thus:

‘We have a problem with our budget. To pay the teachers and so on, we struggle … the teachers are getting a little bit less money and everybody gets a little bit less money.’ (PPS, D, M)

### Implementation of high-quality inclusive education amongst a diversity of learners as per the curriculum

This study explored the participation of learners in all school activities, barriers and problems they faced that impacted their access to the curriculum most especially learners with specific learning difficulties. The results were as follows:

#### Diversity of learners

Some of the schools were using all the available resources to give the students the possibility to learn. They also accommodated a diversity of learners as reported:

‘It is a major challenge to other schools, some other schools can’t accommodate those learners, especially, let’s say, those who may need some extra care.’ (SPS-SEN-CF, H, F)

#### Teaching methods and learning materials

Most of the regular schools did not have the resources to reformat the school materials or to hire special teachers to provide for blind students. In some state schools, they grouped students according to their features so that the ones with disabilities were separated into a different setting thus:

‘We don’t combine them, they study at different hours … because we don’t want to interrupt the others.’ (S-Voc, M, F)

The belief behind this was that the students with disabilities were more easily distracted by outside influences and needed different learning methods. Some students needed more one-on-one attention in order to understand particular topics. To facilitate curriculum access by learners with diverse learning needs, a variety of classroom strategies were used through, modification, adaptations or differentiations.

#### Curriculum adaptation and modification

All the interviewed primary and high schools followed the regular curriculum and therefore developed their very own ways of teaching and trying to reach certain goals within the national standards. They had to incorporate the factor of inclusive education in their concept of teaching all kinds of students the same set of information and skills as required by the government as indicated:

‘We follow the primary school curricula. But because it is a special school, we have our special ways we do prepare the children up to the level, doing the national primary examination.’ (SPS-SEN, H, F)

Those schools that really focused on integrating students with special needs into their classrooms and spreading the message of diversity within their reach seemed to struggle to meet the intended standards in the traditional way. For that reason, most of them had worked out special interventions, which focused on supporting the children, especially in the primary school stage as follows:

‘However much we are following the normal curricula for primary schools … we were allowed by the Ministry of Education to develop a curricula by ourselves, which will be used alone with the national curricula to help our learners, because of their special needs.’ (SPS-SEN, H, F)

Two of the interviewed institution (i.e. a primary school and the centre for vocational training) both mentioned the use of the alternative curriculum. The students in the primary school who were intellectually not capable of participating in all the aspects of the standard curriculum like the rest of the students, the schools had to change to a vocational curriculum, which focused more on practical skills, for example, tailoring as indicated:

‘The bigger percentage of them, after living here … make a living, they get something to do … The biggest problem … most people don’t want to employ them … start their own business. There are so many stereotypes attached to persons with disabilities. We also got a few disappointments … your student begging for money … I met two or three students on the streets. “We don’t give them start up kits, …”’ (S-Voc, M, F)

The vocational training centre did not go through the formal education system but fundamentally focused on teaching hands-on skills to enable young adults with special needs to get jobs and live independent lives thus:

‘We basically give them practical skills … the capacity to stand on their own after failing to go through the formal system.’ (S-Voc, M, F)

Similarly:

‘Those who cannot joint the secondary school, I think we have an option. For example, there is a vocation section.’ (SPS-SEN, H, F)

#### Reaching goals

Basically, all the schools in Uganda were expected to follow the Thematic Curriculum; therefore, teachers were advised to cover certain subjects and learning fields with their students within a set amount of time as required by the National Curricula Development Centre. However, the challenge with the Thematic Curriculum was making sure that a large number of children with different backgrounds were all able to follow along and pass the exams. The schools addressed this challenge in the following ways: One main procedure, which was implemented in nearly all the schools was repeating a certain school level as follows:

‘There is an expected level of performance in the school, for every class we set it to be so minimum, “If you don’t reach the minimum, you would be advised to repeat the class.”’ (SHS, H, M)

Repeating class sometimes made sense because some children needed more time to adjust to the school setting. Alternatively, some students struggled with different subjects and needed to strengthen their knowledge in some fields in order to improve in their school career. Some of the schools described the need of having to repeat a class as some kind of failure because the specific child did not get the result required or:

‘Failed to perform to the expectations.’ (SP, H, F)

This also involved subjecting students to a certain amount of pressure for achieving certain grades and because there were expected levels of performance, students kept that mindset in the back of their heads especially students with disabilities. However, SPS-SEN school was exceptional because it did not focus on achieving higher grades but was committed to providing education to children with disabilities thus:

‘For us, we don’t have to fight for the best grades … We mind about this child doing something … we had seven candidates and they all passed.’ (SPS-SEN, H, F)

#### Teachers’ education and skills

As mentioned previously, the state was responsible for recruiting and funding a certain number of teachers and sending them off to the public and state-aided schools. In order to work as a teacher, the government required them to have a diploma, certificate or a master’s degree from the university or college. The private schools employed teachers themselves because they used part of the tuition to pay them. Usually, teachers in secondary schools basically had two teaching subjects and were specialised in a certain field, whereas teachers in primary education had a wider range of subjects on a less specialised level, as their students were much younger.

Two of the state schools that had a strong foundation on disability (i.e. SPS-SEN and SPS-SEN-CF) were found to have a larger number of staff qualified in the field of special education needs as expressed:

‘So the teachers we have here … about eight teachers, have special training to manage these children. But we have also others, who learnt through experience.’ (SPS-SEN, H, F)

#### Teacher–student ratio

Two of the state schools had over 100 students in a single class. They were closely stacked together and movement between the desks was very difficult as reported:

‘One of the reasons is, the state schools are located far from young children under six years … the state schools often have only one room and one class with 100 pupils and one teacher. If it comes to eating, those schools don’t mind of the child has eaten or not.’ (PPS, D, M)

### Determining the course of action and school routines of private and state schools

#### General daily routines

Every morning (Monday to Friday) when the first morning bell rang, there was a parade for students. The activities included checking student cleanliness and receiving communication from the teachers and student leaders or prefects, before entering their respective classrooms at 08:00 for reading, writing and other classroom work. At 10:30, there was a break of 30 min interval for the children in kindergarten, primary one and two to have breakfast, whilst other students had play. Thereafter, they entered their respective classes for classroom work up to 12:30. Between 12:30 and 14:00 was lunch break. Between 14:00 and 16:30 was classroom work. Between 16:30 and 17:00 was games and sports time. After 17:00 students were released to return home (for day scholars) or return to their dormitories for (boarding students). Both Saturday and Sunday students rest at home or in their respective dormitories. Note that students were free to go for prayers (on Friday, Saturday or Sunday) according to their respective faith.

The study identified that students with special needs were provided with support to enable them cope with the daily school routines, for example, eating breakfast, getting dressed, combing hair, brushing teeth, pushing in wheel-chair, etc. When teaching activities of daily living to children with intellectual disabilities such as toileting, hand-washing, washing clothes, etc., do the following:

‘We keep repeating, reminding them of their routines.’ (SPS-SNE-CF, H, F)

#### Classroom routines

To facilitate curriculum access by learners with diverse learning needs, a variety of classroom strategies were used through, modification, adaptations or differentiations. A lot of effort was provided in giving students with special needs more one-on-one attention. Teachers had to identify their students’ strengths in order to provide the special assistance and guidance throughout the learning process because every child might need a different kind of support as follows:

‘We know the abilities and we can assess the child … We operate like any other primary school, we follow the primary school curricula. But because it is a special school, we have our special ways we do prepare the children up to the level, doing the national primary examinations.’ (SPS-SEN, H, F)

Some schools discussed individual learning goals for certain children who could not follow the basic curriculum because of their disability as follows:

‘Those who are in wheelchairs are ok, their intelligence is normal. But the majority who are able to walk straight … have a lot of learning problems … Some may learn, but at a slow pace. Like we have mild, severe and profound.’ (SPS-SEN, H, F)

Concerning the teaching methods used some schools tried to include everybody in the class and in the school as follows:

‘The usual ones, but it requires special attention in the learning process … use flexible language for the deaf to understand … Our autistic kids need one to one exchange to achieve the learning levels. Many need special guidance or need to be escorted …’ (SPS-SEN-CF, H, F)

The interviewed schools had different positions when it came to separating the students according to their learning level. Some preferred separation as they claimed that they were using different teaching methods for different ‘streams’ and therefore everyone in the group had to get the same needed support and assistance thus:

‘They may be in one room, but when the teacher is giving instructions, he or she handles the children individually, after identifying their strengths. So they may group them according to abilities.’ (SPS-SEN H, F)

However, the students in the ‘higher’ streams got better marks than the students who needed more time with certain topics. Other schools, however, operated in the opposite way by mixing up the students so children from different levels joined one group. Those schools believed in having the students assisting each other so that everyone was able to profit from the united skills. This way, the teacher was not the main focus when supporting the group as the students were able to help each other most times.

The public and state-aided schools had a large number of students per class. Some teachers had to ‘float’ from one class to another because having seven levels in primary school (i.e. Primary 1 to Primary 7) and several streams in each level, teachers had to switch from one learning level to another.

A part from the routine and regular teaching of students, those learners who did not meet the learning goals of the class especially those with dyslexia were supported first through school’s assessment team and later from the government. Whatever their needs, children with dyslexia got assistance from teachers to transcribe or read during lessons and tests. An official had to come from the Uganda National Examinations Board (UNEB) to support these children during the final exams of PLE:

‘We assess and determine the problem of the learner. So when he is in Primary 7, we inform the board in writing … sent people who sat with them in the exam room and they did their exams and passed well.’ (SPS-SEN-CF, H, F)

‘At the school, the way that we support our pupils varies from class to class and from ability to ability.’ (SPS-SEN-CF, H, F)

Where students were all mixed-up, learners from different levels joined one group to enable assisting each other so as to profit from the united skills. This way, the teacher was not the main focus when supporting the group as the students were able to help each other most times.

#### Teaching activities of daily living

All of the schools had specific courses of action regarding their focus, as they did not cater for all kind of impairment. For example, those schools that were founded on the notion of disability also focused on teaching those life skills (i.e. activities of daily living) on top of the set curriculum such as using the bathroom, washing, brushing teeth or even eating and drinking independently. Thus, encouraging the students to live independently whenever possible and getting support when needed or asked for, which essentially promotes their self-confidence thus:

‘When they first come to our school, they have no skill at all. The teacher assesses the child and makes a file … the child cannot use the bathroom alone, brush the teeth, can’t bathe, they were feeding him at home, but now he learned how to feed himself and by the time the parents come, he is more autonomous.’ (SPS-SEN-CF, H, F)

## Discussion

### Understanding of inclusive schools and classrooms

All the six interviewed schools claimed to be catering and working inclusively for children with disabilities. The inclusive practice is more about the right of access, no one left behind, removal of all barriers, embracing a diversity of learners and prevention of exclusion (Rapp & Corral-Granados 2021). Yet, all the six schools in the study excluded students with visual impairments by referring them to attend separate special schools with excuses that the teachers lacked sufficient knowledge and skills for teaching and supporting students with visual impairments. The six schools’ action of discriminating students with visual impairments does not reflect inclusion although they are supported by Belay and Yihun (2020) who argued that special educational services for learners with disabilities are often inadequate in mainstream schools, thus their complete access to the curriculum is hindered because of inadequate teaching skills for modifying or adapting the curriculum, lack of instructional and learning materials such as Braille. Ferreira and Sefotho (2020) also agreed that students with visual impairment need specialised assistance to scholastically participate.

However, the concept of inclusive education is about accepting all learners without exclusion, it is a right based and a route to inclusive societies (EASNIE 2018). Inclusion focuses on diversity or differences in abilities, cultural background and gender as well as how school structures cater for those differences (Sturm 2019). It is also about transforming schools by preventing exclusion so as to provide equal opportunities for a diversity of learners (Ainscow, Slee & Best 2019). UNICEF’s (2017) definition of inclusive education is summarised as follows:

Inclusive education means all children learn together in the same schools (p. 1)

According to Caspersen, Smeby and Olaf Aamodt (2017) ‘inclusion’ covers a variety of school objectives. Yet, the real understanding of inclusive practice by the schools in the study was defined by each school’s concept, the different ways of operation and assessment of children with special needs, as well as the accompanying demands that came with inclusion. There was an obvious distinction between the state and private schools. For instance, as the private institutions demanded some sort of tuition, they had more possibilities to create pleasant learning environment. This included, for example, having smaller classes and more teachers, as well as transport opportunities, appropriate meals and boarding options. In addition, some of the private schools employed therapists and social workers who assisted the students during their school work, especially those who had special needs and required more support. By taking advantage of the given support system, students with special needs participated in regular class more easily and focused on developing their individual strengths whilst gaining more confidence, which is a fundamental component of inclusion because it emphases democracy and social justice (Hernández-Torrano, Somerton & Helmer 2020). Therefore, private schools had a wide range of possibilities to take into account and include students with disabilities into their school because their financial means allowed them to do so. That, however, does not mean that the interviewed state schools were failing to do their job in practicing inclusiveness and high-quality education, as they relied on very limited funds provided by the government. State schools rather had to figure out and trusted the management style of the school principal and sound teaching methods in order to be able to cater for a very large number of students whilst still considering their different needs. As this was extremely challenging and demanded a lot of organisation and knowledge, not all state schools managed to accomplish that task. The state schools also had to follow the theoretical frameworks and political implications of the concept of inclusion in education, which stems from local policies, Hardy and Woodcock (2015) or the United Nations Educational, Scientific and Cultural Organization’s (UNESCO) guidelines for inclusion and ensuring access to education for all (UNESCO 2005). Accordingly, state schools were forced to focus more on the quantity than on the quality because they were obligated to admit all the students who applied because primary education in Uganda was free.

However, even though the schools were acting progressively in many ways, their teaching methods still could not be rated in the inclusive spectrum because they separated their students in each level into different streams according to their ability. Some of these institutions did not believe in this concept and therefore rather separated the students according to their features, so that the ones with disabilities got separated into a different setting. The practicing of separate learning settings might cause the group that is weak to become even weaker (Sarton & Smith 2018). Therefore, understanding that every child has individual strengths and weaknesses, regardless of being age appropriately developed or having a disability is important to consider when catering for everyone and giving every student the opportunity to learn. The exclusion of students with visual and hearing impairment in this study agrees with Pellicano et al. (2017) who found out that exclusion of students with special education needs in schools can take several forms: permanent exclusion (excluded from school altogether) or fixed-term exclusion (short period).

The management of the given funds in state institutions was another controversial topic within the different interviews because the school environments differed a lot from each other especially when considering the aspect of accessibility. Comparing the state primary and secondary schools with the vocational training school all supported by the government and supposedly receiving the same kind of financial support, the study discovered that, whilst one of them was physically accessible for students with physical impairments, other schools did not even provide an appropriate access road. The results agree with Limaye’s (2016) findings that inaccessible physical environment hinder access to education by students with physical disabilities. The study also agrees with Belay and Yihun (2020) that the physical infrastructure such as classrooms, playgrounds and toilets of many state schools are inaccessible for students with disabilities. That led to the question of how such large fluctuations within the different state-supported schools could be explained and tolerated as both parties did not deliver a uniform picture.

### Inclusive education as per the curriculum

Inclusive education provides high-quality education, increased participation, sustainable and systemic change in educational values, cultures and design (UNICEF 2015). The current curriculum, which was incorporated in all Ugandan schools, was called the Thematic Curriculum and was first introduced into the school routines in 2007. The curriculum was put into practice a year after the UNCRPD was adopted and in the same year, as it was signed by Uganda (United Nations Treaty Collection 2018). The new curriculum stood out from all the previous ones, as it proposed innovative learning and teaching methods, emphasised the importance of providing school materials in several different languages and generally shifted from teacher to child-centred teaching structure. This corresponds with Jorgensen et al. (2012) provision that:

Curriculum and instruction are designed to accommodate the full range of student diversity based on universal design principles. Individualized supports are provided to students with significant disabilities to enable them to fully participate and make progress within the general education curriculum …. Presented in a variety of accessible formats …. (p. 6)

As many schools in Uganda struggle with a lack of teachers and a large number of students in one class, schools still try hard to figure out ways to facilitate inclusive education. Teachers in the state schools had to deal with rather large classes (United Nations Development Programme 2016). That made focusing on all the students’ different needs extremely difficult compared with other countries, where the teacher to student ratio is much smaller.

Apart from the vocational training school, the teaching methods of the state schools could not be rated in the inclusive spectrum because they separated their students in each level into different streams according to their ability because of the use of a centralised rigid curriculum. The inflexible instructional methods that permitted only one style of teaching contravened UNESCO’s (2009) principle of an inclusive curriculum. Schuelka (2018) contended that a centralised rigid curriculum, which does not give space for flexibility or modification is unsuitable for inclusive education in schools. The schools claimed to be forced to act that way as they had to deal with a large number of students and that seemed to be the only way for them to able to reach the governmental standards. This streaming subjected students to a competitive situation that was discouraging for students in the lower streams because those in better streams often obtained better marks. This was also the main reason why many families with children with disabilities (especially learning disabilities) tried to get access to private schools to enable their struggling children to follow the teaching and operating methods to avoid dropping out of school. The research findings are similar to a Kenyan study by Disability Africa (2017), which found that the high dropout rates from schools by children with disabilities were caused by multiple barriers that include rigid instructional methods. Other students had to put in a lot of effort during their free time, after school, or during extra lessons, which some of the interviewed state schools offered for free.

#### Changes made by Convention on the Rights of Persons with Disabilities

The CRPD is a fundamental advance step for promoting equal participation of persons with disabilities (PWDs) and its practical effect relies on how court decisions and domestic policies are implemented. The policies and other regulatory frameworks of each member state such as Uganda, act as pillars in guaranteeing inclusion because CRPD provides inclusion guidelines for PWDs and outlines their rights against discrimination and recommends the required support to enable them live independent lives (Bratan et al. 2020). In this regard, the CRPD is a model for future human rights treaties as stated (Kanter 2015):

[*N*]ever before in the history of the UN were the subjects of a treaty invited to play such a prominent role in the drafting process. (p. 8)

Watson and Vehmas (2020) confirmed that the CRPD is upheld by activists and scholars as a ‘paradigm shift’ regarding human rights and disability. Some of CRPD’s critical areas for addressing disability inequality include legal capacity, independent living and reasonable accommodation. Convention on the rights of persons with disabilities has greatly shaped policy and the thinking about disability, Mladenov (2013) and for Uganda, CRPD’s influence brought about a radical change in the disability domain when the *Uganda Persons with Disabilities Act* (UPwDA 2020) was enacted based on CRPD principles. One of UPwDA’s (2020) clauses on non-discrimination in the provision of education services stipulates as follows:

An institution of learning shall not discriminate against a learner with a disability, on the basis of disability. A person who willfully prevents a child with a disability from attaining education commits an offence, and is liable, on conviction, to a fine not exceeding twenty currency points or imprisonment for a term not exceeding six months or both. (pp. 3–4)

The given affirmative action is a great achievement for both the CRPD and PWDs in fighting social discrimination against PWDs in schools of Uganda. In trying to restore the rights of PWDs, the UPwDA (2020), which was adapted from the CRPD, also provides a reference to several items that include respect for the evolving capacities of children with disabilities, accessibility, participation and inclusion, respect for diversity, non-discrimination and equality (Watson & Vehmas 2020). The UPwDA (2020) also calls for sufficient provision of support for individualised services to learners with disabilities and availing enough skilled special needs teachers, thereby anticipating effective and full inclusion of PWDs in the education system in the near future. Basing on the CRPD guiding principles, the World Bank (2018) has been funding Disability-Inclusive Education Programmes in Africa including Uganda. Some of the funding was used in the construction of physically accessible classrooms and accessible ramps in Uganda.

#### The dilemma of deaf inclusion

This study revealed that students with hearing-impairment were struggling with managing the daily conditions at mainstream schools where they appeared unique and often had to pretend that they heard what was being spoken. These mainstream schools had more restrictive environments and were not quite inclusive for deaf learners because they struggled with developing and sustaining social relationships, academic content and lacked a working language for communication. Kompara, Hölbl and Welzer (2021) advocated for the provision of education inclusion in practice and not on paper for traditional minorities like deaf learners in terms of language for communication. In Uganda, like in some developing countries, deaf children begin primary education without a language, and although inclusion would eliminate the discrimination of these children, the reality remains that they lack the language (Khairuddin 2018). Egard, Hansson and Wästerfors (2022) argued that, in addition to lack of language, the loneliness and psychosocial problems experienced by learners with hearing impairment and their increasing poorer academic performance than their peers call for better development and designing of practices that are more inclusive and embracing a diversity of students in schools.

The education of non-deaf children with deaf children as advocated by disability activists is contested by researchers such as, Snoddon and Underwood (2017) who contended that the empowerment and future sustainability of the deaf community will depend on the learning of the sign language by the deaf children and their families and the teachers and other deaf pupils in congregated spaces. Therefore, this sign language is an educational tool, which should be accorded a linguistic status of cultural rights of deaf communities (Khairuddin 2018). Nevertheless, for the deaf community to achieve linguistic status as a cultural right, sign language teaching programmes should be introduced and qualified sign language teachers employed to facilitate sign language accessed by deaf children, their families and non-deaf learners at an equal educational level, (Murray et al. 2018).

Singer and Vroman (2019) also recommended that, instead of concentrating on UNCRPD (2006) least restrictive environment, a more culturally sustaining, liberating and supportive environment should be considered for deaf students. The most suitable educational provisions for deaf children therefore should include easy access to separate classrooms, sign language and assistive devices such as cochlear implants and hearing aids (UNESCO 2020).

Following the given argument there are already existing and contradicting ideologies between the disability rights movements and those of the deaf community, which usually results into disputes (Khairuddin 2018). Whilst the human rights movement considers the deaf community as having the impairment, the deaf community categorically rejects being defined as having a disability or an impairment but instead wish to be regarded as ‘different’ although they enjoy and receive the legal accommodation and benefits meant for PWDs (Harvey 2008). McDonnell (2016) asserted that, although deaf people reject being considered impaired, they share a history of exclusion and other forms of oppression like other PWDs. The major fundamental issues for the deaf community are culture and language and not much to do with cochlea implant, audiograms or decibels whilst their main obstacles include high degree of inequality, inadequate deaf teachers, lack of a working language, inaccessible curricular and inappropriate teaching methods (McDonnell 2016). The denial and rejection of disability by the deaf community whilst enjoying and taking advantage of the legal rights of PWDs also calls for government’s expansion and modification of current laws so that the views of this minority (deaf community) can be accommodated.

#### The dilemma of deaf-blind inclusion

All the schools in the study did not enroll the blind and deaf-blind learners as they claimed not to have the resources for reformatting the school materials or for hiring specialised teachers. In addition, the schools did not know how to effectively communicate with the deaf-blind yet, communication is essential to deaf-blindness, (World Federation of the Deafblind [WFDB] 2018). However, the communication requirements of deaf-blind people are different because of variances in the learnt skills, personal characteristics, sensory impairments, history, type and extent (Hersh 2013). In addition to communication needs, deaf-blind people also have a diversity of other requirements, perceptual motor and cognition development and parent–child relationships (Manga & Masuku 2020). This diversity of requirements can lead to serious educational needs that are difficult to accommodate alone either in special education schools for children with blindness or children with deafness (Wolford 2016). Nelson and Bruce (2016) argued that because learning takes place through hearing and vision, there is a need for a team of paraprofessionals and skilled professionals to design suitable communication and educational opportunities for deaf-blind people. It is necessary to design multiple communication strategies to be put in practice both at school and home environments to facilitate communication for both parties (Manga & Masuku 2020). Accordingly, developing a functional communication system can be essential in the deaf-blind student’s educational progress through participation in social activities, relationship development and facilitating their independence. However, Manga and Masuku (2020) recommend that a functional communication method should be setup to facilitate educational access by learners with deaf-blindness and should involve adequate support and preparedness and possession of appropriate skills and knowledge by educators and their assistants.

### Course of action and school routines

Some school routines of private and state school included supporting the diverse needs of learners, facilitating inclusive classrooms and providing supportive teaching and learning strategies. However, some classes in the two state schools had more than 100 students taught and supervised by one teacher at a time. The classes were too crowded with very little space between desks, which could not permit students using mobility devices to easily navigate. Therefore, this study is in line with Grant et al. (2014) who argued that large class sizes become inaccessible, especially for students with mobility difficulties and hinder participation by all students. Small class sizes are very suitable for students’ learning, especially those with learning difficulties and can improve interaction between the individual student and the teacher especially on a one-to-one basis (Ballantine & Spade 2014:82). The teacher is also in a better position to address the specific needs of the students and use diverse instructional approaches flexibly.

The study discovered that, when working with students with intellectual disabilities you have to keep repeating, reminding them of their routines, for example, washing hands, getting dressed, combing hair, brushing teeth and toileting. Remember to always provide a lot of feedback and support for the student for each step in the routine and to build the student’s independence by reducing the verbal reminders. Procedures and routine in school especially when supporting students with intellectual disabilities were found to be very fundamental as they enabled students to get organised and somehow become independent (Hayes et al. 2018).

## Conclusion

The understanding of inclusion within the six different schools was mainly characterised by the awareness of the students’ needs, the accessibility aspect, the use of different educational or teaching methods and the overall school concept. The study finally concluded that, although all the schools were struggling in practicing inclusiveness, state-aided special primary school for children with physical disabilities (SPS-SEN) and state-aided special primary school church founded (SPS-SEN-CF), which were founded on the background of disability performed better than either private primary school (PPS) or any other state primary school (SPS), state high school (SHS) or state vocational training school (S-Voc). Whilst welcoming and teaching students of different kinds despite their impairments, all the schools showed significant differences when it came to admission, tuition, accommodation and ways of operating. This study revealed that the schools were missing a realistic and achievable standard required for inclusive practice. The study therefore recommended that the Ugandan MOES should carry out an assessment and evaluation of schools on a regular basis in order to achieve the implementation of unified regulations within each and every school. It should be observed that the provision of an access ramp alone or the admission of one particular category of students is not enough to qualify a school as being inclusive. The study therefore recommended additional educational materials and adequate funding for schools as the base of a functioning and well-organised education system so as to facilitate free and high-quality public education for all students, including those with special needs. Finally, the study recommended that, in order to fully achieve UN’s inclusive education, all stakeholders (including policymakers and implementers) should put in place a combination of the following factors: (1) positive attitude, non-exclusion and admission for all, provide barrier-free and accessible physical environment, appropriate, teaching and learning materials; (2) skilled teachers in the area of special education needs; (3) involvement of parents, government, donor agencies and civil society organisations; (4) a flexible curriculum; (5) adequate funding; (6) availability of assistive devices; (7) non-institutionalisation; (8) engaging professionals and other stakeholders; and (9) free education for all and addressing the specific educational needs of the diversity of learners.

## References

[CIT0001] African Union, 2014, *An outlook on education report: Southern African development community*, Association for the Development of Education in Africa, Tunis Belvedere, viewed 20 May 2021, from https://www.adeanet.org/sites/default/files/au_outlook_report_sadc_english_version_2014_web.pdf

[CIT0002] Ainscow, M., Slee, R. & Best, M., 2019, ‘The Salamanca statement: 25 years on’, *International Journal of Inclusive Education* 23(7–8), 671–676. 10.1080/13603116.2019.1622800

[CIT0003] Ballantine, J.H. & Spade, J.Z., 2014, *Schools and society: A sociological approach to education*, Sage, London.

[CIT0004] Belay, M.A. & Yihun, S.G., 2020, ‘The challenges and opportunities of visually impaired students in inclusive education: The case of Bedlu’, *Journal of Pedagogical Research* 4(2), 112–124. 10.33902/JPR.2020060437

[CIT0005] Bratan, T., Fischer, P., Maia, M. & Aschmann, V., 2020, ‘Implementation of the UN Convention on the Rights of Persons with Disabilities: A comparison of four European countries with regards to assistive technologies’, *Societies* 10(4), 1–25. 10.3390/soc10040074

[CIT0006] Busetto, L., Wick, W. & Gumbinger, C., 2020, ‘How to use and assess qualitative research methods’, *Neurological Research and Practice* 2(14), 1–10. 10.1186/s42466-020-00059-z 33324920 PMC7650082

[CIT0007] Caspersen, J., Smeby, J.C. & Olaf Aamodt, P., 2017, ‘Measuring learning outcomes’, *European Journal of Education* 52(1), 20–30. 10.1111/ejed.12205

[CIT0008] Chitiyo, A. & Dzenga, C.G., 2021, ‘Special and inclusive education in Southern Africa’, *Journal of Special Education Preparation* 1(1), 55–66. 10.33043/JOSEP.1.1.55-66

[CIT0009] Disability Africa, 2017, *Kenya*, viewed 09 May 2022, from https://www.disability-africa.org/kenya

[CIT0010] Egard, H., Hansson, K. & Wästerfors, D., 2022, *Accessibility denied: Understanding inaccessibility and everyday resistance to inclusion for persons with disabilities*, Routledge, New York, NY.

[CIT0011] European Agency for Special Needs and Inclusive Education (EASNIE), 2018, *Evidence of the link between inclusive education and social inclusion: Literature review*, EASNIE, Odense, viewed 27 May 2021, from https://www.european-agency.org/resources/publications/evidence-literature-review

[CIT0012] Ferreira, R. & Sefotho, M.M. (eds.), 2020, ‘Understanding Education for the Visually Impaired’, in Opening Eyes Volume 1, pp. i–414, AOSIS, Cape Town.

[CIT0013] Grant, L., Stronge, J., Xu, X., Popp, P., Sun, Y. & Little, C., 2014, *West meets East: Best practices from expert teachers in the U.S. and China*, Association for Supervision and Curriculum Development Publishing, Alexandria.

[CIT0014] Hands 4 Uganda, 2018, *Did you know: Education*, viewed 22 March 2021, from https://hands4uganda.org/did-you-know-education/

[CIT0015] Hardy, I. & Woodcock, S., 2015, ‘Inclusive education policies: Discourses of difference, diversity and deficit’, *International Journal of Inclusive Education* 19(2), 141–164. 10.1080/13603116.2014.908965

[CIT0016] Harvey, E.R., 2008, ‘Deafness: A disability or a difference’, *Health Law & Policy* 2(1), 42–57.

[CIT0017] Hayes, A.M., Dombrowski, E., Shefcyk, A. & Bulat, J., 2018, *Learning disabilities screening and evaluation guide for low- and middle-income countries*, RTI Press Publication, Research Triangle Park, NC. 31449375

[CIT0018] Hernández-Torrano, D., Somerton, M. & Helmer, J., 2020, ‘Mapping research on inclusive education since Salamanca statement: A bibliometric review of the literature over 25 years’, *International Journal of Inclusive Education* 26(9), 893–912. 10.1080/13603116.2020.1747555

[CIT0019] Hersh, M., 2013, ‘Deafblind people, communication, independence, and isolation’, *Journal of Deaf Studies and Deaf Education* 18(4), 446–463. 10.1093/deafed/ent022 23749484

[CIT0020] Initiative for Social and Economic Rights (ISER), 2015, *Privatisation, discrimination and the right to education in Uganda*, viewed 27 February 2021, from https://www.iser-uganda.org/images/downloads/privatisation_discrimination_and_right_to_education.pdf.

[CIT0021] Jorgensen, C.M., McSheehan, M., Schuh, M. & Sonnenmeier, R.M., 2012, *Essential best practices in inclusive schools*, University of New Hampshire, National Center on Inclusive Education, Institute on Disability/UCEDD, Durham, viewed 16 March 2021, from https://www.tash.org/wp-content/uploads/2011/03/Essential-Best-Practices-070312-FULL-Jorgensen.pdf

[CIT0022] Kanter, A., 2015, *The development of disability rights under international law: From charity to human rights*, Routledge, Abingdon.

[CIT0023] Khairuddin, K.F., 2018, ‘The inclusion of deaf children in Malaysian primary schools: Experiences of parents, staff members and children’, School of Education, Environment and Development, PhD thesis, University of Manchester.

[CIT0024] Kim, J., 2021, ‘An analysis of Uganda’s vocational education: Assessing human capital and human development approaches’, *Issues in Educational Research* 31(2), 556–573.

[CIT0025] Kompara, M., Hölbl, M. & Welzer, T., 2021, *Communication challenges in inclusive education faced by deaf and non-deaf people*, viewed 04 May 2022, from https://www.unisiegen.de/zew/insign/outcomes/

[CIT0026] Limaye, S., 2016, ‘Factors influencing the accessibility of education for children with disabilities in India’, *Global Education Review* 3(3), 43–56.

[CIT0027] Mahlo, D., 2017, ‘Rethinking inclusive education in an African context’, in N. Phasha, D. Mahlo & G.J.S. Dei (eds.), *Inclusive education in African contexts: Anti-colonial educational perspectives for transformative change*, pp. 101–113, Sense Publishers, Rotterdam.

[CIT0028] Manga, T. & Masuku, K.P., 2020, ‘Challenges of teaching the deaf-blind learner in an education setting in Johannesburg: Experiences of educators and assistant educators’, *South African Journal of Communication Disorders* 67(1), a649. 10.4102/sajcd.v67i1.649 PMC7343947 32633989

[CIT0029] McDonnell, P., 2016, ‘Disability, deafness and ideology in the late twentieth and early twenty-first centuries’, *Educação & Realidade, Porto Alegre* 41(3), 777–788. 10.1590/2175-623661091

[CIT0030] Ministry of Education and Sports (MOES), 2021, *Schools and institutions calendar*, MOES, Kampala.

[CIT0031] Ministry of Education and Sports (MOES), 2017, *Education and sports sector strategic plan 2017–2020*. Uganda, viewed 12 June 2022, from https://www.globalpartnership.org/content/education-and-sports-sector-strategic-plan-2017-2020-uganda

[CIT0032] Ministry of Education and Sports (MOES), 2018, *Special needs and inclusive education department*, viewed 12 June 2022, https://www.education.go.ug/special-needs-inclusive-education/

[CIT0033] Mladenov, T., 2013, ‘The UN Convention on the Rights of Persons with Disabilities and its interpretation’, *European Journal of Disability Research* 7(1), 69–82. 10.1016/j.alter.2012.08.010

[CIT0034] Murray, J.J., Snoddon, K., De Meulder, M. & Underwood, K., 2018, ‘Intersectional inclusion for deaf learners: Moving beyond general comment no. 4 on article 24 of the United Nations Convention on the Rights of Persons with Disabilities’, *International Journal of Inclusive Education* 24(7), 691–705. 10.1080/13603116.2018.1482013

[CIT0035] Mutebe, J., 2017, *The government’s role in private education in Uganda*, viewed 10 March 2021, from https://www.hopeforlifekatanga.com/blog/its-time-for-the-government-to-regulate-private-schools-and-institutions-of-learning

[CIT0036] Mwesigwa, A., 2015, ‘Uganda’s success in universal primary education falling apart’, *The Guardian*, viewed 06 May 2021, from https://www.theguardian.com/global-development/2015/apr/23/uganda-success-universal-primary-education-falling-apart-upe.

[CIT0037] Nelson, C. & Bruce, S.M., 2016, ‘Critical issues in the lives of children and youth who are deafblind’, *American Annals of the Deaf* 161(4), 406–411. 10.1353/aad.2016.0033 27818397

[CIT0038] Pellicano, E., Brede, J. Remington, A., Kenny, L. & Warren, K., 2017, *Excluded from school: Autistic students’ experiences of school exclusion and subsequent re-integration into school*, Sage, London.

[CIT0039] Rapp, A.C. & Corral-Granados, A., 2021, ‘Understanding inclusive education – A theoretical contribution from system theory and the constructionist perspective’, *International Journal of Inclusive Education*. 10.1080/13603116.2021.1946725

[CIT0040] Sarton, E. & Smith, M., 2018, *The challenge of inclusion for children with disabilities – Experiences of implementation in Eastern and Southern Africa*, Disability Inclusion, UNICEF Think Piece Series: UNICEF Eastern and Southern Africa Regional Office, Nairobi.

[CIT0041] Schuelka, M.J., 2018, *Implementing inclusive education. K4D helpdesk report*, Institute of Development Studies, Brighton.

[CIT0042] Singer, S. & Vroman, K., 2019, ‘A new model of educating deaf students’, in D. Ford (ed.), *Keywords in radical education*, pp. 90–105, Koninklijke Brill Publishers, Amsterdam.

[CIT0043] Snoddon, K. & Underwood, K., 2017, ‘Deaf time in the twenty-first century: Considering rights frameworks and the social relational model of deaf childhood’, *Disability and Society* 32(9), 1400–1415. 10.1080/09687599.2017.1320269

[CIT0044] Sturm, T., 2019, ‘Constructing and addressing differences in inclusive schooling–comparing. Cases from Germany, Norway and the United States’, *International Journal of Inclusive Education* 23(6), 656–669. 10.1080/13603116.2018.1444105

[CIT0045] Tenny, S., Brannan, G.D., Brannan, J.M. & Sharts-Hopko, N.C., 2021, *Qualitative study*, StatPearls Publishing, Treasure Island, FL.

[CIT0046] Uganda Bureau of Statistics, 2019, *Statstical Abstract*, UBOS Publications, Kampala, viewed 11 May 2021, from https://www.ubos.org/wpcontent/uploads/publications/01_20202019_Statistical_Abstract_-Final.pdf

[CIT0047] Uganda Persons with Disabilities Act (UPwDA), 2020, *Persons with Disabilities Act*, Uganda Government Gazette, Kampala.

[CIT0048] United Nations Convention on the Rights of Persons with Disabilities (UNCRPD), 2006, *Convention on the Rights of Persons with Disabilities and optional protocol*, viewed 27 January 2021, from https://www.un.org/disabilities/documents/convention/convoptprot-e.pdf

[CIT0049] United Nations Convention on the Rights of Persons with Disabilities (UNCRPD), 2010, *Uganda’s Initial Status Report*, viewed 27 January 2021, from https://rodra.co.za/images/countries/uganda/concluding_observations/Uganda%20Initial%20Status%20Report%20to%20the%20UNCRPD.pdf.

[CIT0050] United Nations Educational, Scientific and Cultural Organization (UNESCO), 2005, *Guidelines for inclusion: Ensuring access to education for all*, UNESCO, Paris.

[CIT0051] United Nations Educational, Scientific and Cultural Organization (UNESCO), 2009, *Policy guidelines on inclusion in education*, Paris, viewed 27 January 2022, from https://unesdoc.unesco.org.

[CIT0052] United Nations Educational, Scientific and Cultural Organization (UNESCO), 2020, *Global Education Monitoring Report 2020: Inclusion and Education - All Means All*, UNESCO Publishing, Paris.

[CIT0053] United Nations Children’s Fund (UNICEF), 2015, *The investment case for education and equity*, UNICEF, New York, viewed 15 February 2021, from https://www.unicef.org/reports/investment-case-education-and-equity

[CIT0054] United Nations Children’s Fund (UNICEF), 2017, *Inclusive education: Including children with disabilities in quality learning: What needs to be done?*, viewed 15 February 2021, from https://www.unicef.org/eca/sites/unicef.org.eca/files/IE_summary_accessible_220917_brief.pdf

[CIT0055] United Nations, 2016, *Concluding observations on the initial report of Uganda*, Committee on the Rights of Persons with Disabilities, viewed 24 April 2016, from https://digitallibrary.un.org/record/830776?ln=en#record-files-collapse-header.

[CIT0056] United Nations Development Programme, 2016, *Human development reports: Uganda*, viewed 24 April 2021, from http://hdr.undp.org/en/countries/profiles/UGA.

[CIT0057] United Nations Treaty Collection, 2018, ‘Status of treaties’, *Human Rights*, viewed 24 April 2021, from https://treaties.un.org/pages/Treaties.aspx?id=4&subid=A&lang=en

[CIT0058] Watson, N. & Vehmas, S., 2020, *Disability and human rights*, Routledge, London. 32543798

[CIT0059] Wolford, M., 2016, *A school psychologist’s guide to deafblindness: Identifying & supporting students with combined hearing-vision loss*, Ohio Center for Deafblind-Education, viewed 03 May 2021, from https://www.ohiodeafblind.com/tools-and-resources/ocdbe-products/6-a-school-psychologist-s-guide-to-deafblindness-identifying-supporting-students-with-combined-hearing-vision-loss-2016/file

[CIT0060] World Atlas, 2019, *Maps of Uganda*, viewed 04 February 2021, from https://www.worldatlas.com/webimage/countrys/africa/uganda/ugland.htm.

[CIT0061] World Bank, 2018, *Disability-inclusive education in Africa program*, viewed 14 February 2021, from https://www.worldbank.org/en/topic/disability/brief/disability-inclusive-education-in-africa-program.

[CIT0062] World Education Services (WES), 2020, *Education in Uganda*, viewed 19 February 2022, from https://wenr.wes.org/2020/10/education-in-uganda.

[CIT0063] World Federation of the Deafblind (WFDB), 2018, *Global Report 2018*, viewed 07 May 2022, from https://wfdb.eu/wfdb-report-2018/wfdb-conclusion/.

[CIT0064] Zubairi, A. & Rose, P., 2019, *Equitable financing of secondary education in sub-Saharan Africa*, viewed 19 February 2022, from https://mastercardfdn.org/wp-content/uploads/2019/03/SEA-Finance-Equity_REAL_Final-Version_Feb-2019-1.pdf

